# Study of a Liquid Plug-Flow Thermal Cycling Technique Using a Temperature Gradient-Based Actuator

**DOI:** 10.3390/s141120235

**Published:** 2014-10-27

**Authors:** Yusuke Fuchiwaki, Hidenori Nagai

**Affiliations:** 1 Health Research Institute, National Institute of Advanced Industrial Science and Technology (AIST), 2217-14, Hayashi-cho, Takamatsu, Kagawa 761-0395, Japan; 2 CEA-LETI, Minatec Campus, 17 rue des Martyrs, 38054 Grenoble Cedex 9, France; 3 Health Research Institute, National Institute of Advanced Industrial Science and Technology (AIST), 1-8-31 Midorigaoka, Ikeda, Osaka 563-8577, Japan; E-Mail: hide.nagai@aist.go.jp

**Keywords:** thermal cycling, DNA amplification, PCR, microfluidics

## Abstract

Easy-to-use thermal cycling for performing rapid and small-volume DNA amplification on a single chip has attracted great interest in the area of rapid field detection of biological agents. For this purpose, as a more practical alternative to conventional continuous flow thermal cycling, liquid plug-flow thermal cycling utilizes a thermal gradient generated in a serpentine rectangular flow microchannel as an actuator. The transit time and flow speed of the plug flow varied drastically in each temperature zone due to the difference in the tension at the interface between temperature gradients. According to thermal distribution analyses in microfluidics, the plug flow allowed for a slow heating process, but a fast cooling process. The thermal cycle of the microfluid was consistent with the recommended temperature gradient for PCR. Indeed, amplification efficiency of the plug flow was superior to continuous flow PCR, and provided an impressive improvement over previously-reported flow microchannel thermal cycling techniques.

## 1. Introduction

During the influenza pandemic in 2009, an accurate diagnosis using conventional technology required at least several hours [1]. To quickly curtail an outbreak, rapid field detection of the target genes is needed. To meet this demand, flow channel-based polymerase chain reaction (PCR) microfluidic devices are seen as systems that could provide rapid detection in the field. Since a flow channel-based PCR microfluidics device was first reported by Martin *et al.*, in the late 1990s [2], interest has grown in an automated PCR system that can perform all the assay steps on a single chip. In principle, PCR reagents are loaded into a long micro-flow channel and repeatedly carried through individual temperature zones for denaturation, extension and annealing. This approach has several advantages, such as: (1) the heater does not require strict temperature control; (2) rapid PCR amplification is possible due to rapid thermal cycling; and (3) it is easier to automate routine work such as injections, DNA amplification and detection [3–9]. In addition, this approach can be applied to reverse-transcription PCR (RT-PCR) by integrating the microfluidic reverse-transcription (RT) process on a single chip, as demonstrated by the rapid diagnosis of influenza.

In continuous flow channel-based PCR, the system is composed of several temperature zones and a long micro-flow channel to carry PCR reagents to individual temperature zones, enabling high speed DNA amplification free from the influence of the heat transfer coefficient of the chamber material. However, most of the previously-reported flow channel-based PCR systems have some practical problems, for example, requiring external flow control equipment, continuous flow of large amounts of PCR reagents into the microchannel, and a need to circumvent the destabilization of flow caused when bubbles are generated in the denaturation zone as the temperature approaches the boiling point of water. Normally, it is necessary to increase the internal pressure of the microchannel in continuous flow channel-based PCR by pre-containing the viscous liquid to prevent bubble formation in the denaturation zone [10]. Since these processes are not so simple, the entire setup necessary for continuous-flow PCR experiments often becomes cumbersome.

Our aim is to develop a practical flow channel-based PCR system that achieves fast DNA amplification simply by a droplet injection. In this study, we focused on utilizing the thermal gradient generated in a continuous serpentine microchannel as the actuator to perform plug-flow DNA amplification. The small amount of PCR solution passes rapidly through the area where air bubbles typically form before they are generated and destabilize the flow. This approach allows for fast and simple flow channel-based PCR operation.

## 2. Experimental Section

### 2.1. Reagents and Materials

The following reagents for PCR were obtained from a CycleavePCR Core kit from TaKaRa (Otsu, Japan): 10 × CycleavePCR buffer, dNTP mixture (2.5 mM), Mg solution (25 mM), positive control primer mix, positive control probe, Tli RNase H II (200 U/μL), Takara Ex Taq HS (5 U/μL), and dH_2_O. The positive controls (1 μL of a plasmid containing 10,000 templates) in the Cycleave PCR Core kit were used as the target genes for all PCR analyses. The optimum sequence size for amplification is given as 100–150 bp. This kit employs Cycling Probe Technology, a highly sensitive detection method using a combination of chimera probes (composed of RNA and DNA) and RNase H. The specific sequence of the target gene can be detected efficiently after amplification, with the amplification efficiency being estimated from the obtained fluorescence intensity.

COP ZEONEX 480 (Tg of COP 480: 138 °C) was purchased from Zeon Corporation (Tokyo, Japan). COP is a 2-mm-thick thermoplastic biocompatible polymer. The characteristics of this heat-resistant resin are comparable to PC and PS (polystyrene). COP is superior for containing aqueous solutions due to its low water absorption coefficient and small nonspecific adsorption to fluidic channels. Pressure-sensitive adhesive (PSA) film (model number: 9795) was purchased from CS-LABO (Japan). The temperature of the channel close to the moving fluid can be analyzed since it is very thin; consequently, PSA film allows uniform bonding and exhibits thermotolerant properties.

### 2.2. Fabrication of Flow-Through PCR Microfluidics Device

The micro-flow channel on the COP substrate was fabricated by cutting microchannels with a numerical control (NC) machine (Micro MC-2, PMT Corporation, Fukuoka, Japan), which was automatically operated using CAD programs. A milling cutter was used to create a ball-end mill with radius 200 μm. The cutting feed rate was one millimeter per second and the spinning rate was 8000 revolutions per second. The overall length of the microchannel (300 μm in depth and 400 μm in width) was approximately 2.8 m (40 cycles × 0.07 m per cycle).

The micro-channel was enclosed by simply applying PSA film to the cyclo-olefin polymer (COP) substrate. PSA film allows uniform bonding and exhibits thermotolerant properties. Although the fabrication and proper use of most microfluidic chip devices requires extensive technical knowledge, experience, and skill, this fabrication process of the flow channel PCR chip device is relatively straightforward and simple.

**Figure 1.**
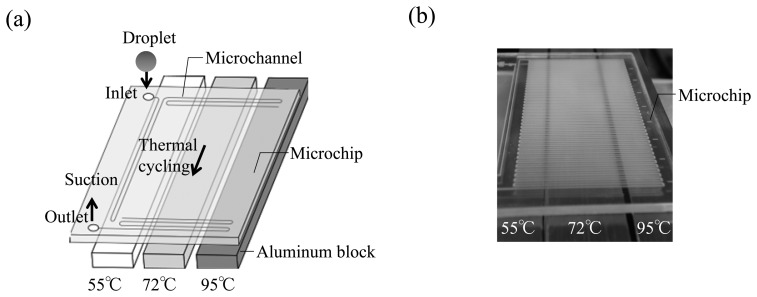
A flow-through PCR microfluidics device made by applying pressure-sensitive adhesive (PSA) film on cyclo-olefin polymer (COP) substrate: (**a**) Diagrammatic illustration of liquid plug-flow PCR microfluidic device; (**b**) Photo of the flow-through PCR microfluidic device.

The microfluidic device was designed to perform 40 thermal cycles for amplification. The PCR chip device was 80 mm long and 50 mm wide. The heater and cooler were composed of 15 × 10 × 100 mm aluminum blocks. The heat-transfer aluminum block contained a heat conductor and temperature sensor (Kyushu-Nissho Co., Fukuoka, Japan). The aluminum block for cooling was in contact with a Peltier cooling element (SPE-UC-100, E.H Sakaguchi Corporation, Tokyo, Japan). The chip device was placed on the aluminum blocks, and the PCR solution containing the target genes was introduced through the inlet with a syringe pump. The blocks were configured such that the PCR solutions passed through three different temperature zones repeatedly (Figure 1a,b). The aluminum block temperatures for extension and denaturation were set at 72 and 95 °C, respectively. The optimum temperature for the annealing zone was considered to be less than 60 °C. The PCR products were collected in microplate wells and their fluorescence intensities were detected with a fluorescence microplate reader (Fluoroskan Ascent, Thermo Scientific, Yokohama, Japan). The moving fluid was visualized by IR imaging using a thermal imager (Ti10, Fluke Corporation, *WA*, USA).

### 2.3. Features of Liquid Plug-Flow System and Continuous Flow System

**Figure 2.**
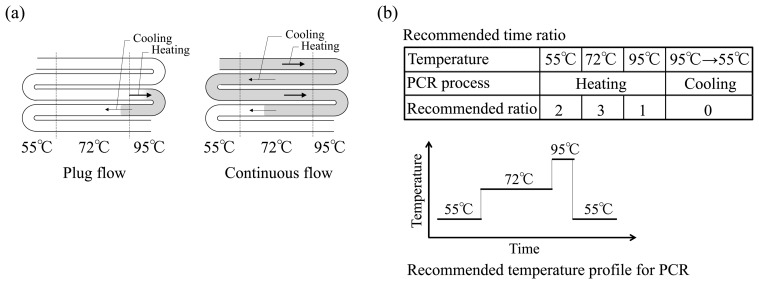
Methodology for flow-through PCR using a microfluidics system: (**a**) Liquid plug-flow and continuous flow; (**b**) Recommended time profile for PCR.

**Figure 3.**
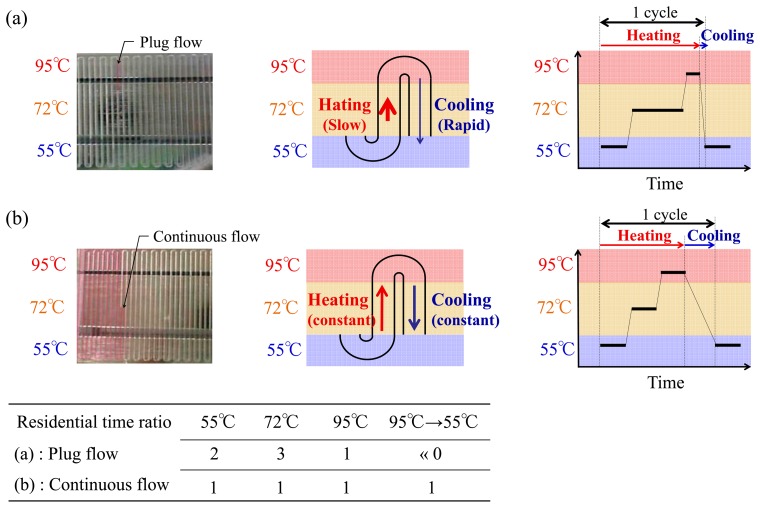
Features of the thermal cycle based on the liquid plug-flow and continuous flow in serpentine rectangular flow microchannel: (**a**) Plug flow-based thermal cycling technique presented by using a temperature gradient between 95 °C and 55 °C; (**b**) Continuous flow-based thermal cycling.

As a simple approach to resolving the problems associated with continuous flow PCR systems mentioned above, liquid plug-flow PCR (1 μL) was attempted as a more practical alternative (Figure 2a). Utilizing the difference in tension at the interface between the denaturation (95 °C) and annealing (55 °C) zones in the microchannel was studied first. In general, the time ratio typically recommended for PCR is 2:3:1 for annealing (55 °C): extension (72 °C): denaturation (95 °C) (Figure 2b). It is preferable to reduce the time for the cooling process because extended cooling increases the risk of residual product formation, such as dimer formation. Indeed, the liquid plug-flow system exhibited a significant difference from the continuous flow system in terms of the transit time and flow rate (Figure 3a,b). The plug flow allowed for slow heating from 55 °C to 95 °C, but also had fast cooling back to 55 °C. This difference is due to the change of the interface tension between temperature gradients, making flow-through PCR more simple and automatic.

Plug-flow minimizes the risk of destabilization of the flow because the entire liquid plug passes the point at which bubble generation generally occurs before any bubble can be dislodged from its nucleation point. Furthermore, the plug-flow devices proposed here only require the PCR reagents in the plug-liquid to be introduced to the microchannel (Figure 1a), and then the fluid flow is expected to promote agitation caused by internal convection. Thus, increased mixing efficiency is achieved. In addition, DNA amplification was not observed when the liquid-plug volume was more than 1 μL. This is because the plug flow could not act as a thermal gradient for an actuator.

## 3. Results and Discussion

### 3.1. Transit Time Analysis in Each Temperature Zone

The tight-sealing performance of the PSA film prevents any liquid plug loss by evaporation, even if the liquid plug is heated close to boiling in the 95 °C zones. In a liquid plug flow-based thermal cycling system, the entire liquid plug passes the point at which bubble generation generally occurs before any bubble can be released from its generation point. However, if the flow speed is insufficient, air bubbles will be released, resulting in unsuccessful DNA amplification due to destabilization of the fluidic flow. To obtain the optimum time ratios for PCR, transit time analysis of the plug-flow liquid is very important for successful DNA amplification.

**Figure 4.**
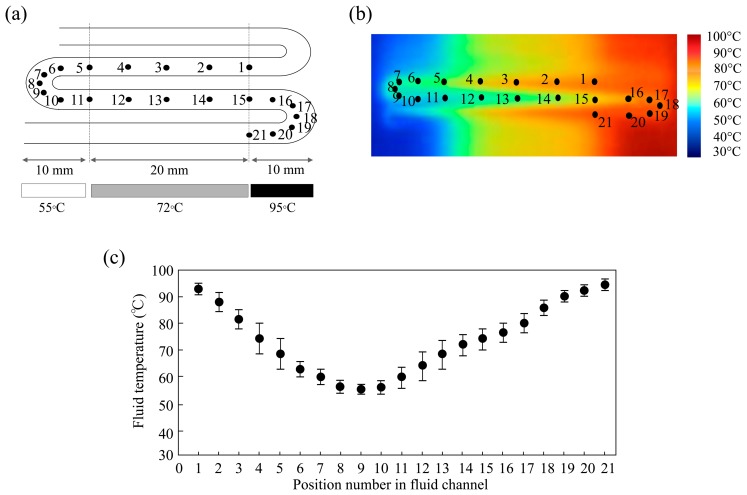
Temperature analysis at points marked on the PSA film and temperature distribution along the microchannel during flow: (**a**) Measuring points along the microchannel; (**b**) Measuring points for infrared imaging when the solution is flowing in the microchannel; (**c**) Fluid temperature distribution along the position number in fluid channel. All measurements were taken three times for each position number.

The temperature distribution along the PSA film was investigated using IR imaging. The denaturation, extension and annealing temperatures were set to 95, 72 and 55 °C, respectively. IR imaging of the thinner PSA film (thickness: 100 μm) allowed analysis of the temperature close to the microfluid itself (Figure 4). The measuring points were marked along the microchannel as shown in Figure 4a,b. The thermal distribution in the microfluidics could be profiled by IR imaging, as shown in Figure 4c, and the conditions for efficient PCR amplification were investigated. Deviations in the temperature distribution were approximately 1.0–7.7 °C, which fall in the allowable range. In addition, the deviations in the continuous flow-method were approximately 0.7–6.8 °C. It was found that the temperature distribution was approximately the same even if the plug flow created a flowing rhythm due to the temperature gradient. The temperature profiling indicated that liquid plug-flow (15 s per cycle) provides a temperature gradient recommended for PCR.

**Figure 5.**
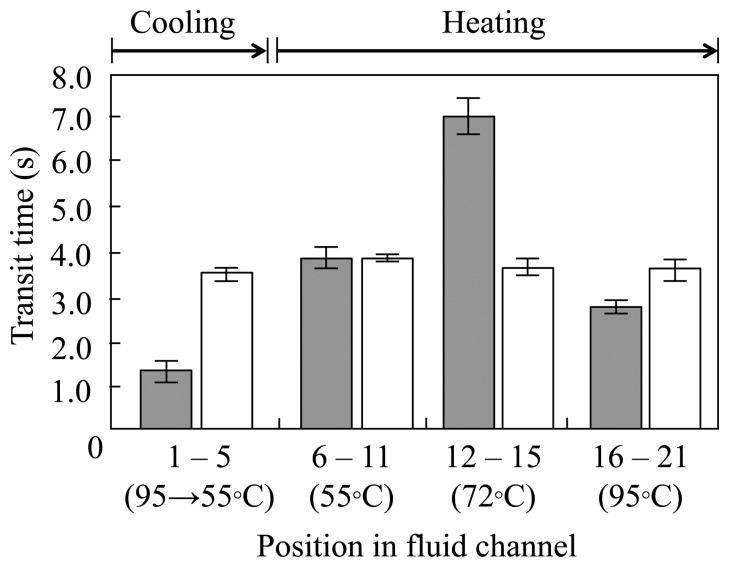
Transit time in each temperature zone. Thermal cycle was performed in 15 s per cycle. The gray and white bars show the transit time of plug flow and continuous flow, respectively. All measurements were taken three times for each position in fluid channel.

Next, the transit time in each temperature zone was investigated and compared to continuous flow (Figure 5). The transit time was longest between measuring points 11–15. An increased extension zone is very beneficial for PCR amplification. In contrast, the transit time was the shortest between measuring points 1–5. These results indicate that the heating time in the extension zone was longer than anticipated, but the cooling time from denaturation to annealing was shorter. The time ratio typically recommended for PCR amplification is 2:3:1 for annealing:extension:denaturation. In general, the cooling process from denaturation to annealing in flow-through PCR requires moving the fluid as quickly as possible. This is because the denatured single-stranded DNA is very likely to form double-stranded bonds with the template strand or the complementary fragments in this process, resulting in the reduction of DNA amplification efficiency.

### 3.2. Amplification Efficiency

**Figure 6.**
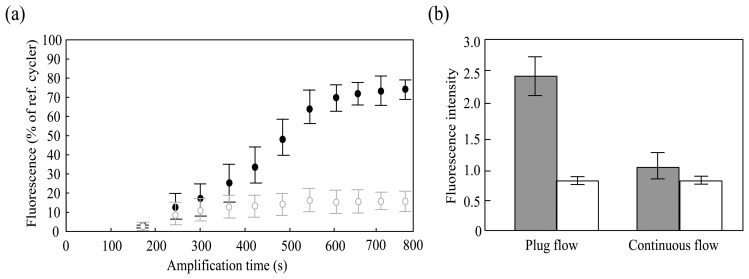
Amplification efficiency by the plug flow-based thermal cycling technique. (**a**) Relationship of PCR amplification yield to the thermal cycler and cycling time. The closed and open circles represent the positive and negative controls, respectively. One microliter of a fluid element passes through the microchannel for 40 cycles. All measurements were taken three times for each cycling time; (**b**) Comparison of plug-flow system and continuous-flow with a total cycling time of 600 s for 40 cycles. The gray and white bars show the positive and negative controls, respectively. All measurements were taken three times for each condition.

Yields of PCR amplification compared to those with thermal cyclers were investigated against cycling time (Figure 6a). The values were normalized to the fluorescence of the product from the standard thermal cycler as a reference and plotted against the total cycling time. Significantly, a flow time of 600 s showed a fluorescence of 67% compared to the conventional thermal cycler (commercial PCR equipment), while that of the negative control not containing target genes was less than 10%. The amplified product was also detected with sufficient sensitivity. Fluorescence increased as a sigmoidal function, which is a typical curve for PCR amplification. Overall, the fluorescence intensity obtained with the plug-flow chip was lower than that observed with the conventional thermal cycler. Because the long micro-channel allows for adsorption of PCR components to the surface of the chip due to the large surface area-to-volume ratio of the plug, there is a risk of a decrease in the amplification efficiency with micro-channel-based systems compared to conventional thermal cyclers.

Adsorption of PCR components was one of the main reasons for the lower fluorescence intensity observed here. Amplification efficiency by liquid plug-flow PCR was compared to conventional continuous flow-through PCR. The DNA amplification efficiency for plug-flow PCR was found to be approximately 2.5 times that of continuous flow-through PCR with a total cycling time of 600 s for 40 cycles (Figure 6b). Previously, it was difficult to obtain high amplification using plug-flow PCR because the flow speed, temperature gradient, and bubble generation must be continuously adjusted to that of the PCR recommended conditions. However, the plug-flow technique in this study made it possible to utilize the difference in tension at the interface between temperature gradients as an actuator, leading to the recommended PCR condition with only pumping force and heating. Furthermore, this technique not only minimizes residual PCR products but also ensures a long heating time at the extension zone. Consequently, the fluorescence intensity of the plug flow technique exhibited a significant increase compared to conventional continuous flow PCR, thus making this PCR technique more practical.

## 4. Conclusions

We developed a practical flow channel-based PCR system that achieves fast DNA amplification simply by a droplet injection. A liquid plug-flow thermal cycling was accomplished using a temperature gradient-based actuator. The transit time and flow speed of the plug flow varied drastically in each temperature zone due to the difference in tension at the interface between temperature gradients. According to the thermal distribution analyses in microfluidics, the plug flow allowed for a slow heating process from 55 °C to 95 °C, but also a fast cooling process back to 55 °C. The present method created a thermal cycle of the microfluid consistent with the recommended temperature gradient for PCR. Indeed, amplification efficiency of the plug flow was superior to continuous flow PCR. To date, flow-through thermal cycling has been performed by a continuous flow method, a complex operation requiring large amounts of PCR reagents. On the other hand, this method uses only suction by the pumping force in the outlet port and a simple droplet injection of the sample reagents into the inlet. Thus, this flow-through PCR chip would make a significant impact in areas such as clinical applications and the reduction of bioterrorism threats.
